# Health Inequity in Georgia During the COVID-19 Pandemic: An Ecological Analysis Assessing the Relationship Between County-Level Racial/Ethnic and Economic Polarization Using the ICE and SARS-CoV-2 Cases, Hospitalizations, and Deaths in Georgia as of October 2020

**DOI:** 10.1089/heq.2021.0118

**Published:** 2022-03-15

**Authors:** Amit Eichenbaum, Allan D. Tate

**Affiliations:** ^1^ College of Veterinary Medicine, University of Georgia, Athens, Georgia, USA.; ^2^ Epidemiology and Biostatistics, College of Public Health, University of Georgia, Athens, Georgia, USA.

**Keywords:** COVID-19, income inequality, racial inequality, residential segregation

## Abstract

**Introduction:**

The coronavirus disease 2019 (COVID-19) pandemic disproportionately burdens communities of color in the United States. The prevalence of preexisting conditions in these populations has not accounted for the observed health inequities. A growing body of research indicates a significant role of racialized residential segregation and income inequality on health outcomes. The Index of Concentration at the Extremes (ICE) is a metric which captures socio-spatial and economic polarization that has proven to be a valuable predictor of a large variety of health outcomes.

**Objectives:**

The primary objective of this ecologic study was to determine the impact of socio-spatial and economic segregation on severe acute respiratory syndrome coronavirus 2 (SARS-CoV-2) morbidity and mortality in Georgia.

**Methods:**

The ICE scores for racial/ethnic, economic, and racialized economic segregation for each county in Georgia (*n*=159) were calculated and investigated as predictors of increased SARS-CoV-2 positivity rate, case-hospitalization rate, and case-mortality rate after controlling for the prevalence of preexisting conditions (diabetes, obesity, and smoking) and potential barriers to care (uninsured rate).

**Results:**

Counties with the largest income disparity had 1.57 times the case rate (*p*<0.0001) and 1.7 times (*p*<0.01) the case-mortality rate compared to the most privileged counties. Cases in counties with the largest racialized economic segregation were 1.8 times more likely to be hospitalized (*p*<0.0001).

**Conclusion:**

Racialized economic segregation is a strong correlate of pandemic health inequities in Georgia and highlights the need for structural interventions to address barriers to minority and vulnerable population health. Increased focus and efforts to address the structural and systematic barriers faced by communities of color is necessary to address health inequities.

## Introduction

By fall of 2020, the United States had become the global epicenter of the coronavirus disease 2019 (COVID-19) pandemic, leading the world in both the number of cases and deaths.^1^ As demographic data regarding novel-coronavirus cases and outcomes became available, severe health inequities in COVID-19 morbidity and mortality rapidly became evident.^2–5^ Indigenous, Black, and Latinx communities experienced a disproportionate burden of cases, hospitalizations, and deaths, with mortality rates from COVID-19 nearing or exceeding three times that of white, non-Hispanic Americans.^6,7^ These trends have remained consistent with those reported during the H1N1 pandemic in 2009.^8,9^

The widely utilized conceptual model developed by Blumenshine et al. describes three factors that impact the development of health disparities during an influenza pandemic: differential exposure, susceptibility for disease, and disparities in receiving timely and effective treatment once disease has developed.^10,11^ This framework, although developed in the context of an influenza pandemic, is transportable to respiratory illnesses like severe acute respiratory syndrome coronavirus 2 (SARS-CoV-2) and the COVID-19 pandemic.

Preexisting conditions, including respiratory and cardiovascular impairments, that represent one component of the communicable disease framework yet have been insufficient alone to explain racial/ethnic and socioeconomic health disparities observed during the H1N1 and COVID-19 pandemics.^12–15^ Evidence suggests that, instead, the structural determinants of health, like disproportionately high exposures and limited access to care, are likely the stem of observed health disparities in a pandemic.^9,13^

The disproportionate burden of disease on communities of color during the COVID-19 pandemic has been described with dominant attention toward individual-level preventive interventions, as is consistent with the majority of health disparity interventions.^16^ A consequence of this point of view is that individual characteristics such as prevalence of preexisting health conditions were erroneously thought to explain such health disparities. It has become increasingly clear that racial and ethnic health disparities are fundamentally caused by the impact of structural racism (i.e., policies, institutions, and practices), which in turn dictate the distribution, or lack thereof, of resources, opportunities, and power.^9,12–15,17–20^

Quantifying the consequences of systematic barriers on health is challenging, and traditional metrics utilized for population studies, such as the Gini-coefficient for income inequality and the Index of Dissimilarity for residential racial segregation, are limited in their utility for measuring social and economic polarization that may influence how groups access health resources across numerous pathways. Another is the Theil entropy index, which operationalizes disordered and ordered states relative to an ideal reference state to characterize social stratification.^21^

Methods for the quantitative assessment of the consequences of structural racism have been advanced by Krieger et al., the adjusted Index of Concentration at the Extremes (ICE), which conveys inequitable group relationships between society groups at suitable geographic dimensions.^22,23^ This feature makes ICE a suitable proxy for characterizing population exposure to structural racism.^17^ It is also a useful measure to test the weak income inequality hypotheses that postulate that low privilege affords less health advantages compared to most privileged groups.^24^

Identifying evidence-based structural interventions to disassemble barriers to health that are upstream of the individual require that quantitative assessments conceptualize research questions with measures that accurately reflect structural risk.

Evidence supports that the ICE is a valuable predictor of health outcomes, with findings indicating that the ICE was a more sensitive metric to health inequities compared to other common measures like poverty rate.^17,23,25–28^ There is a growing body of public health research which uses this metric, but to the authors’ knowledge, the ICE measure has not been applied in the state of Georgia for any health outcomes and this study is the first to utilize ICE to monitor COVID-19 population health in Georgia.

As the ICE measure captures structural, not individual, social determinants of health, utilization of the metric at the county level is likely to better represent the impact of systematic inequality on group outcomes appropriate for quantifying expected gains that could be achieved with structural interventions. Future pandemics are inevitable, and thus, new preventive measures are needed to interrupt socially determined risk during emerging infectious disease outbreaks.^29^

The current study describes the distribution of racial and income inequity across Georgia counties and examines how county-level features of racial/ethnic and economic polarization have affected novel-coronavirus infection, hospitalization, and mortality in Georgia by October 2020. This study advances an increasingly convincing argument that social polarization accelerates emerging infectious disease incidence and widens health disparity gaps.

New preventive strategies are needed to reduce transmissibility and improve care delivery for the most under-resourced groups to control emerging epidemics. Attention to how these structures impose excess population risk is likely to improve not only high-risk population health but also the health outcomes of more privileged groups during infectious disease outbreaks.

## Methods

### Study population

The current ecologic study summarized individual level data to the county-level data for the state of Georgia (*N*=159 counties, *N*=10,297,484 residents) drawn from the U.S. Census American Community Survey (ACS) between 2014 and 2018.

### Exposures: ICE and poverty measures, county-level predictors, and controls

The Index of Concentration at the Extremes (ICE) is a metric that was designed to reveal the degree to which county residents cluster on privilege and deprivation geospatially (i.e., county).^23^ The values range from −1 to 1, with −1 indicating 100% of the population being concentrated in deprivation, while 1 indicates that 100% of the population is concentrated in privilege.^26^ Values around 0 do not necessarily represent equitable privilege, rather that the geospatial unit is near the compositional center (similar neighbor traits).^26^

The Index of the Concentration of Extremes^22^ is computed as follows:


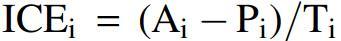


Where:

A_i_=number of individuals in the group of privilege

P_i_=number of individuals in the group of deprivation

T_i_=total population of interest in the geographic area (e.g., county).

Three ICE scores were calculated, including ICE for income, ICE for race, and ICE for combined income and race.

(1) ICE for income utilizes the extremes of the ACS household income categories 20th and 80th household income percentile, or those with an income greater than or equal to $100,000 and those with an income less than or equal to $20,000, respectively.
(2) ICE for race/ethnicity utilizes those who self-identify as non-Hispanic Black and non-Hispanic white and the total non-Hispanic or Latino population.
(3) The combined ICE for income+race/ethnicity compares non-Hispanic White persons whose household income was greater than or equal to the 80th income percentile and non-Hispanic Black persons whose household income was less than or equal to the 20th income percentile.^23,25^ The income cutoffs are the same as those used to calculate ICE for income, being those with an income greater than or equal to $100,000 and those with an income less than or equal to $20,000.

The ICE score may be utilized as a continuous variable or categorical measure. For use in this study, ICE scores were converted into a categorical measure by grouping ICE scores into tertiles for interpretability. The ICE scores in the first tertile, or T1, consist of those with the lowest privilege or highest deprivation. The ICE scores in the third tertile, or T3, consist of those with the highest privilege. The second tertile, T2, consists of those scores which fall in the middle.

Geographic units with ICE center values close to 0 have been found to be due to a low concentration of individuals at either extreme, rather than an equal number of individuals in each extreme.^28^ Those with center values close to 0 indicate that common compositional characteristics are shared by neighbors within the geographic unit, rather than there being an equal number of individuals in each extreme.^28^ As an additional metric and control, poverty percentile was calculated utilizing data from the ACS. Tertiles were calculated and implemented for the poverty percentile as well.

**FIG. 1.**
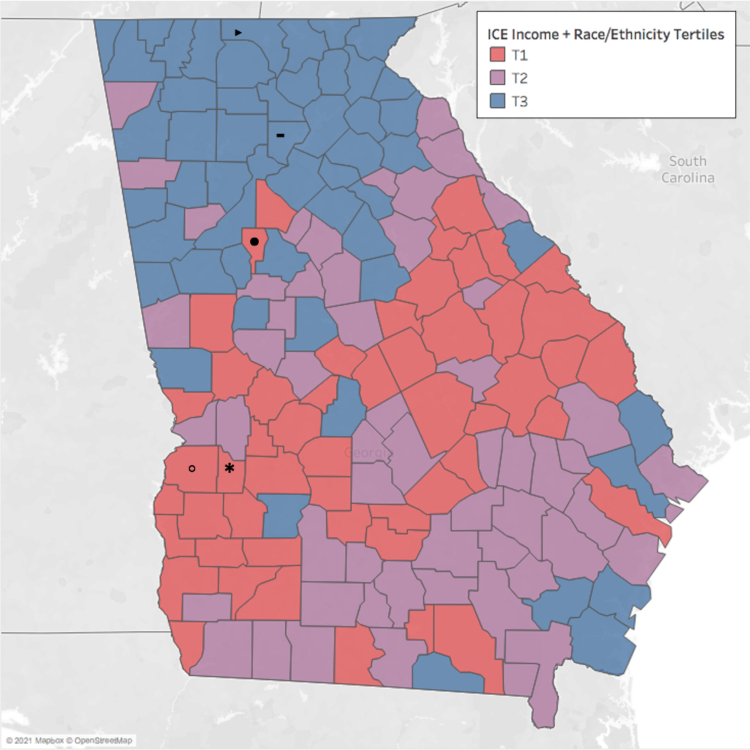
Tertile Distribution for the ICE for Income+Race/Ethnicity, Georgia, 2020. The ICE values are calculated using data sources from the Census Bureau ACS 2014–2018 5-year annual average. Sample size is *n*=159 counties in Georgia. ICE for income+race/ethnicity compares low-income non-Hispanic Black American versus high-income non-Hispanic White American. The ICE scores in the first tertile (T1) consist of those with the lowest privilege, while those in the third tertile (T3) consist of those with highest privilege. Highlighted counties: • Clayton County, ▸ Fannin County, ⁃ Forsyth County, ○ Stewart County, * Webster County. ACS, American Community Survey; ICE, Index of Concentration at the Extremes.

Figure 1 demonstrates the distribution of tertiles for the combined ICE for income+race/ethnicity across the counties in Georgia (*n*=159).

### Outcomes: COVID-19 cases, hospitalizations, and deaths

Data on COVID-19 cases and health outcomes for the State of Georgia are sourced from the publicly available dataset from the Georgia Department of Public Health Daily Status Report. Novel coronavirus cases reported by the state are PCR-confirmed cases, and the data utilized are cumulative since the start of collection on March 02, 2020. Analysis was conducted utilizing the publicly available dataset from the Georgia Department of Public Health which reported cumulative PCR-confirmed COVID-19 cases (*n*=307,955), hospitalizations (*n*=28,599), and deaths (*n*=7083) through October 7, 2020. Each of these outcomes was standardized to a population size per 100 people at the county level for interpretability.

### Statistical analysis

In the current ecologic study, exploratory descriptive statistics was computed and plotted using maps to examine county clustering patterns. Inferential statistical procedures were used to quantify determinants of COVID-19 morbidity and mortality at the county-level unit of analysis. General linear models were fitted with a log link and poisson variance family to examine relative burden due to ICE tertiles (operationalized as a categorical variable). Models include Huber-White robust standard errors to deal with misspecification of the error term and to adjust for multiple confounding of the county-level confounders described above.

Predicted marginal rates were used to quantify absolute burden, and relative rates (RRs) against the privileged reference group (i.e., ICE tertile 3) were used to describe social gradients of the polarization predictors. *A priori*, overall statistical significance was set to *P*<0.05 for all inferential tests. Multiplicity adjustments were not incorporated as pairwise statistical testing was not performed as a primary analytic aim. Controls for the percent of adults with diabetes, percent of adult smokers, percent of uninsured adults (under the age of 65), and percent of obese adults were included in adjusted analyses. All data management and analysis were performed in Stata 16.1MP (College Station, TX).

## Results

The ICE score representative of racial/ethnic segregation (high values indicate privileged counties on the measure) ranged from −0.57 (Clayton County) to 0.95 (Fannin County). The ICE that characterizes income+racial/ethnic segregation ranged from −0.35 (Stewart County) to 0.39 (Forsyth County). The ICE scores for race/ethnicity have the largest range, indicating that this variable captures the greatest dimension of concentrations at extremes. The poverty percentile ranges from 5.6% in Fannin County to 41% in Webster County. The map presented in Figure 1 demonstrates the clustering of tertiles associated with the ICE for income+race/ethnicity and highlights the aforementioned counties.

### ICE as a determinant of county COVID case rate

Counties in the least privileged ICE tertile (T1) were positively associated with an increased COVID-19 case rate for all representations of racial/ethnic and economic segregation (Table 1). The strongest relationship was seen in counties with the largest income disparity, as measured by ICE for income, having 1.57 times the case rate compared to the most privileged tertile, T3 (95% confidence interval [CI]=1.17–1.89, *p*=0.0008). This resulted in a case rate of 4.78 cases per 100 individuals in counties occupying the lowest tertile compared to 3.04 cases per 100 individuals in counties occupying the most privileged tertile, an additional 1.74 cases per 100 individuals.

**Table 1.** Positive SARS-CoV-2 Case Rate Per 100 People: SARS-CoV-2 Rates and RRs in Relation to the ICE and the Poverty Percentile, Georgia, 2020

	ICE income^a^			ICE race/ethnicity^b^			ICE income+race/ethnicity^c^			Poverty		
ICE tertile^d^	Rate	RR (95% CI)	Overall ***p*** value	Rate	RR (95% CI)	Overall ***p*** value	Rate	RR (95% CI)	Overall ***p*** value	Rate	RR (95% CI)	Overall ***p*** value
T1 (low)	4.78	1.57 (1.17–1.89)	0.0008	4.42	1.49 (0.84–1.38)	0.0053	4.79	1.69 (0.98–1.61)	0.0011	3.28	0.94 (1.04–1.53)	0.0063
T2	3.26	1.07 (1.00–1.59)		3.75	1.26 (1.18–2.09)		3.55	1.25 (1.26–2.27)		4.41	1.26 (0.76–1.16)	
T3 (high)	3.04	1 (Ref.)		2.97	1 (Ref.)		2.83	1 (Ref.)		3.49	1 (Ref.)	

Models adjusted for prevalence of adult diabetes, adult smokers, uninsured adults, and obese adults. Data for the calculation of ICE and Poverty Percentiles sourced from the Census Bureau ACS 2014–2018 5-year annual average. Sample size is *n*=159 counties in Georgia. PCR-confirmed positive COVID-19 cases per 100 people, standardized by county size, cumulative data from March 02, 2020 to October 07, 2020.

^a^ Low income versus high income.

^b^ Non-Hispanic Black American versus non-Hispanic White American.

^c^ Low-income non-Hispanic Black American versus high-income non-Hispanic White American.

^d^ ICE Tertile cut points −1=highest concentration at deprivation, 1=highest concentration of privilege. ICE for income (low income vs. high income): T1−1 to −0.22; T2−0.23 to less than −0.12; T3−0.13 to 1. ICE for race/ethnicity (non-Hispanic Black American vs. non-Hispanic White American): T1−1 to <0.21; T2 0.22 to <0.51; T3 0.51 to 1. ICE income+race/ethnicity (low-income non-Hispanic Black American vs. high-income non-Hispanic White American): T1−1 to less than −0.042; T2−0.043 to <0.074; T3 0.075 to 1. Poverty (percentile): T1 0.25 to 1; T2 0.19 to <0.24; T3 0 to <0.18.

CI, confidence interval; COVID-19, coronavirus disease 2019; ICE, Index of Concentration at the Extremes; RR, relative rate; SARS-CoV2, severe acute respiratory syndrome coronavirus 2.

The poverty measure exhibited a nonlinear pattern where counties in the top and bottom tertiles experience similar absolute risk, with counties with the highest concentration of poverty having a rate ratio of 0.94 (95% CI=0.98–1.61, *p*=0.0063) compared with the least poverty concentration.

### ICE as a determinant of county case hospitalization rate

Counties occupying the least privileged tertile were positively associated with excess COVID-19 case hospitalization for all ICE measures (*p*<0.0001) (Table 2). The absolute case-hospitalization rate in counties occupying the lowest tertile of the combination ICE for income and race was 14.11 hospitalizations per 100 cases, compared to 7.84 hospitalizations per 100 cases. Counties in the lowest tertile for each measure experienced an additional 4 to 6 hospitalizations per 100 cases compared to those in the most privileged tertile. Counties occupying the least privileged ICE tertiles for income, race/ethnicity, and combined income and race/ethnicity experienced 1.49, 1.54, and 1.8 times the case-hospitalization rate, respectively, compared to the most privileged tertile (Table 2).

**Table 2.** COVID-19 Hospitalization Rate Per 100 Positive Cases: COVID-19 Rates and RRs in Relation to the ICE and the Poverty Percentile, Georgia, 2020

	ICE income^a^			ICE race/ethnicity^b^			ICE income+race/ethnicity^c^			Poverty		
ICE Tertile^a^	Rate	RR (95%CI)	Overall ***p*** value	Rate	RR (95% CI)	Overall ***p*** value	Rate	RR (95%CI)	Overall ***p*** value	Rate	RR (95% CI)	Overall ***p*** value
T1 (low)	12.33	1.49 (1.25–1.78)	<0.0001	13.14	1.54 (1.34–1.77)	<0.0001	14.11	1.80 (1.51–2.14)	<0.0001	10.71	1.05 (0.933–1.19)	0.69
T2	10.73	1.30 (1.12–1.50)		9.63	1.13 (0.98–1.29)		9.75	1.24 (1.07–1.45)		10.36	1.02 (0.90–1.15)	
T3 (high)	8.25	1 (Ref.)		8.53	1 (Ref.)		7.84	1 (Ref.)		10.17	1 (Ref.)	

Models adjusted for prevalence of adult diabetes, adult smokers, uninsured adults, and obese adults. Data for the calculation of ICE and Poverty Percentiles sourced from the Census Bureau ACS 2014–2018 5-year annual average. Sample size is *n*=159 counties in Georgia. Hospitalization of PCR-confirmed positive COVID-19 cases per 100 people, standardized by county size, cumulative data from March 02, 2020 to October 07, 2020.

^a^ Low income versus high income.

^b^ Non-Hispanic Black American versus non-Hispanic White American.

^c^ Low-income non-Hispanic Black American versus high-income non-Hispanic White American.

^d^ ICE Tertile cut points −1=highest concentration at deprivation, 1=highest concentration of privilege. ICE for income (low income vs. high income): T1−1 to −0.22; T2−0.23 to less than −0.12; T3−0.13 to 1. ICE for race/ethnicity (non-Hispanic Black American vs. non-Hispanic White American): T1−1 to <0.21; T2 0.22 to <0.51; T3 0.51 to 1. ICE income+race/ethnicity (low-income non-hispanic Black American vs. high-income non-hispanic White American): T1−1 to less than −0.042; T2−0.043 to <0.074; T3 0.075 to 1. Poverty (percentile): T1 0.25 to 1; T2 0.19 to <0.24; T3 0 to <0.18.

### ICE as a determinant of county case-mortality rate

Counties occupying the least privileged tertile for all ICE measures were positively associated with elevated COVID-19 mortality rates (Table 3). Higher concentrations of income inequality were the strongest predictor of elevated mortality, with 3.86 deaths per 100 cases compared to 2.27 deaths per 100 cases in the most privileged counties (RR=1.70; 95% CI=1.22–2.37; *p*=0.006). In these counties with more significant income disparity, SARS-CoV-2 cases were 1.7 times more likely to end in a mortality, meaning an additional 1.59 persons died per 100 cases of COVID-19. On average, counties in the lowest tertile had one more death per 100 cases compared to those in the top tertile.

**Table 3.** COVID-19 Case Mortality Per 100 People: COVID-19 Rates and RRs in Relation to the ICE and the Poverty Percentile, Georgia, 2020

	ICE income^a^			ICE race/ethnicity^b^			ICE income+race/ethnicity^c^			Poverty		
ICE tertile^d^	Rate	RR (95% CI)	Overall ***p*** value	Rate	RR (95%CI)	Overall ***p*** value	Rate	RR (95% CI)	Overall ***p*** value	Rate	RR (95% CI)	Overall ***p*** value
T1 (low)	3.86	1.70 (1.22–2.37)	0.006	3.42	1.39 (1.07–1.83)	0.046	3.59	1.52 (1.09–2.13)	0.05	2.93	1.00 (0.79–1.23)	0.5922
T2	2.94	1.29 (0.98–1.72)		3.15	1.29 (0.99–1.66)		3.05	1.29 (0.98–1.71)		3.23	1.10 (0.89–1.37)	
T3 (high)	2.27	1 (Ref.)		2.45	1 (Ref.)		2.36	1 (Ref.)		2.93	1 (Ref.)	

Models adjusted for prevalence of adult diabetes, adult smokers, uninsured adults, and obese adults. Data for the calculation of ICE and Poverty Percentiles sourced from the Census Bureau ACS 2014–2018 5-year annual average. Sample size is *n*=159 counties in Georgia. Mortality of PCR-confirmed positive COVID-19 cases per 100 people, standardized by county size, cumulative data from March 02, 2020 to October 07, 2020.

^a^ Low income versus high income.

^b^ Non-Hispanic Black American versus non-Hispanic White American.

^c^ Low-income non-Hispanic Black American versus high-income non-Hispanic White American.

^d^ ICE Tertile cut points −1=highest concentration at deprivation, 1=highest concentration of privilege. ICE for income (low income vs. high income): T1−1 to −0.22; T2−0.23 to less than −0.12; T3−0.13 to 1. ICE for race/ethnicity (non-Hispanic Black American vs. non-Hispanic White American): T1−1 to <0.21; T2 0.22 to <0.51; T3 0.51 to 1. ICE income+race/ethnicity (low-income non-Hispanic Black American vs. high-income non-Hispanic White American): T1−1 to less than −0.042; T2−0.043 to <0.074; T3 0.075 to 1. Poverty (percentile): T1 0.25 to 1; T2 0.19 to <0.24; T3 0 to <0.18.

## Discussion

This county-level ecological study provides evidence that socio-spatial and economic polarization exacerbates COVID-19 disease burden in Georgia. The results of this study are overall consistent with the literature, with three outcomes—case rate, case-hospitalization rate, and mortality rate—being significantly associated with the ICE measure and additional predictors after controlling for the prevalence of preexisting conditions (diabetes, obesity, and smoking) and potential barriers to care (uninsured rate).

Our findings showed that counties in Georgia with the most polarization experienced greater coronavirus morbidity and mortality, suggesting that structural interventions administered at the county level may be needed to address health inequities during emerging infectious disease outbreaks. Overall, these findings support the weak income inequality hypothesis, which proposes that unequal distribution of resources and privilege contributes to negative health outcomes.^24^

Successful structural interventions first require understanding the contexts in which health disparities occur.^30^ Structural barriers that prevent a community's ability to adhere to public health guidelines, like social distancing, may be responsible for the increased risk in case rate and case hospitalization. African Americans are more likely to work front line, “essential” jobs which increase exposure, are less likely to have paid sick days, and are 60% more likely to be uninsured than white workers.^31^ Black workers are also more likely to live in densely populated housing and in multigenerational households.^31^ It is likely a combination of all of these factors which contribute to counties with greater deprivation and polarization experiencing increased COVID-19 morbidity.

Access to care is another likely causal factor. An analysis of hospital visits across seven states revealed that black patients were six times less likely to get coronavirus testing or related treatment than white patients.^32^ This is consistent with previous studies, which indicate that white Medicaid patients were three times more likely to be prescribed antiviral treatment for seasonal influenza.^33^

It is in this context that, despite the fact that black patients are more likely to be denied both testing and treatment, there is still an increased case rate and case-hospitalization rate in communities with racial/ethnic residential and economic segregation. The prevalence of this relationship in the face of controlling for preexisting conditions and uninsured rate signals a potentially very meaningful relationship between a county's polarization and COVID-19 health outcomes.

The U-shaped gradient (Table 1) demonstrated between the poverty measure and case rate, where counties in the top and bottom tertiles experience similar absolute risk, may be explained by potentially decreased SARS-CoV-2 testing in areas with poverty percentiles above 19% (T1), which are more likely to be occupied by Black Americans. Access to testing (and now vaccinations) is more concentrated in wealthier whiter neighborhoods.^34,35^ Against the background of every other outcome measured in this study demonstrating significant burden at the middle tertile, the case rate stands out as being sensitive to testing availability. This may indicate that the case rate should not be used as an early indicator of burden in emerging disease outbreaks as it is likely a biased measure.

Testing availability is amenable to intervention and should be over-resourced to populations that stand to generate community spread. In addition, the lack of evidence supporting the poverty measure as a predictor of health outcomes suggests that utilizing such a measure may not be prudent at the county level.

The findings of this study are strengthened by the utilization of the ACS, a reliably collected and comprehensive measure of social and economic stratification measured prior to the start of the pandemic (2014–2018). The end-point of the data in October minimizes seasonal trends and the impact of holiday traveling and congregation on the data. The sourcing of more than 7 months of cumulative case, hospitalization, and COVID-19 mortality data from Georgia Department of Public Health provided accurate, publicly available reproducible data about the experience of a serious emerging pandemic in a state with a history of racial/ethnic and economic segregation.

Utilization of county-level geographic units for analysis provides an additional strength, as the state of Georgia's regional healthcare system is broken into 14 units for which the discrete mutually exclusive members of each healthcare coalition region are counties.^36^ Ecologic studies can be sensitive to measurement bias on the geographic unit of choice (the modifiable areal unit problem). While it has been argued that utilization of small spatial units introduces less bias, county-level operationalization is most consistent with the structural environment of the provisioning and care delivery system in Georgia.^37,38^ County-level units of analysis may be the best theoretical level inference for quantifying structural disadvantage that affects minoritized populations during emerging pandemics.

The structural racism measure (ICE) is increasingly being adopted as a valid measure of social polarization. The findings of this study cohere with the body of research support about ICE as a valuable predictor of population health gradients. Finally, the adjustment for multiple county-level confounders minimized a number of theoretical sources of data-driven bias about the relationship between structural determinants of health.

The current study had limitations related to the nature of the pandemic. The cross-sectional nature of the data captures counties at different stages of the pandemic. Decomposing the time dimension and analyzing time trends over months of the pandemic may reveal new information about how to intervene to prevent spread. This information has implications for the conservation of resources, as underserved communities may experience peaks of burden earlier or later than the privileged.

In addition, all outcomes are likely impacted by low capture of asymptomatic cases at the time of data collection. Geospatial epidemiologic methods are needed to account for how populations travel for care when they reside in a county without a clinical infrastructure. A consequence of this feature likely resulted in more conservative estimation of the strength of the county-level structural determinants of hospitalization. Information on county of residence is not available through the Georgia Department of Public Health Daily Status Report, although it is reported to the Department of Health. Future studies should consider using information regarding at-risk individual's proximity to testing and care to better understand the trends in counties without clinical infrastructure.

### Health equity implications

These findings contribute to the growing body of research which supports that it is racism, not race, which is responsible for the devastating impact of COVID-19 on Indigenous, Black, and LatinX communities in the United States. These findings demonstrate that residential racial/ethnic segregation is a significant predictor of disproportionate pandemic disease burden. Structural interventions are needed to interrupt health disparities caused by structural racism. Such interventions should be designed to maximize adherence and minimize population burden in complying with such requirements. They should also be resourced so that material costs of compliance are not on individuals or households.

Focus on racial/ethnic and economic polarization as targets for public health interventions to mitigate health disparities may prove to be valuable in alleviating the impact of future emerging infectious disease epidemics for the total population. Our results indicate that the utilization of the ICE in public health monitoring of health inequities in the State of Georgia is feasible and may reveal additional targets for resource allocation and interventions to reduce health disparities during this pandemic and the next.

## Availability of Data and Materials

The datasets used and/or analyzed during the current study are available from the corresponding author on reasonable request.

## Abbreviations Used

ACS: American Community Survey
CI: confidence interval
COVID-19: coronavirus disease 2019
ICE: Index of Concentration at the Extremes
RR: relative rate
SARS-CoV-2: severe acute respiratory syndrome coronavirus 2
